# Human olfactory neural progenitor cells reveal differences in IL-6, IL-8, thrombospondin-1, and MCP-1 in major depression disorder and borderline personality disorder

**DOI:** 10.3389/fpsyt.2024.1283406

**Published:** 2024-04-09

**Authors:** Alan Patrick Davalos-Guzman, Francisco Javier Vegas-Rodriguez, Gerardo Bernabe Ramirez-Rodriguez, Monica Flores-Ramos, Perla Vanessa Romero-Luevano, Jorge Julio Gonzalez-Olvera, Ricardo Arturo Saracco-Alvarez

**Affiliations:** ^1^ Laboratorio de Neurogénesis, Subdirección de Investigaciones Clínicas, Instituto Nacional de Psiquiatría Ramón de la Fuente Muñiz, Ciudad de México, Mexico; ^2^ Subdirección de Investigaciones Clínicas, Instituto Nacional de Psiquiatría “Ramón de la Fuente Muñiz”, Ciudad de México, Mexico

**Keywords:** major depression, borderline personality disorder, olfactory epithelium, adult neurogenesis, progenitor cells, soluble factors

## Abstract

**Background:**

Discovering biological markers is essential for understanding and treating mental disorders. Despite the limitations of current non-invasive methods, neural progenitor cells from the olfactory epithelium (hNPCs-OE) have been emphasized as potential biomarker sources. This study measured soluble factors in these cells in Major Depressive Disorder (MDD), Borderline Personality Disorder (BPD), and healthy controls (HC).

**Methods:**

We assessed thirty-five participants divided into MDD (n=14), BPD (n=14), and HC (n=7). MDD was assessed using the Hamilton Depression Rating Scale. BPD was evaluated using the DSM-5 criteria and the Structured Clinical Interview for Personality Disorders. We isolated hNPCs-OE, collected intracellular proteins and conditioned medium, and quantified markers and soluble factors, including Interleukin-6, interleukin-8, and others. Analysis was conducted using one-way ANOVA or Kruskal-Wallis test and linear regression.

**Results:**

We found that hNPCs-OE of MDD and BPD decreased Sox2 and laminin receptor-67 kDa levels. MASH-1 decreased in BPD, while tubulin beta-III decreased in MDD compared to controls and BPD. Also, we found significant differences in IL-6, IL-8, MCP-1, and thrombospondin-1 levels between controls and MDD, or BPD, but not between MDD and BPD.

**Conclusions:**

Altered protein markers are evident in the nhNPCs-OE in MDD and BPD patients. These cells also secrete higher concentrations of inflammatory cytokines than HC cells. The results suggest the potential utility of hNPCs-OE as an *in vitro* model for researching biological protein markers in psychiatric disorders. However, more extensive validation studies are needed to confirm their effectiveness and specificity in neuropsychiatric disorders.

## 1 Introduction

Depression and borderline personality disorder have significant morbidity and psychosocial disability burden (1, 2). One of the main difficulties in the diagnosis and treatment of mental disorders is the shortage of biomarkers that allow for objective and replicable measurements, taking as a definition that a biomarker is a characteristic that is objectively measured and serves as an indicator of normal biological processes, pathological processes, or response to therapeutic interventions (3). Interestingly, one of the most studied sources of samples in the search for biomarkers of psychiatric disorders is serum (4). However, some researchers have pointed to the relevance of the olfactory epithelium (OE) for searching biomarkers for psychiatric disorders (5), mainly because the OE is a neurogenic zone in which the generation of neurons occurs constitutively during the adult stage, presenting a marked decline during aging (6). As in the hippocampus, the generation of new neurons in the OE occurs from stem/progenitor cells, which proliferate, migrate, differentiate, and survive to give rise to newborn neurons. However, the generated neurons in the OE are olfactory sensory neurons which are part of the olfactory system (7). Thus, neuroplastic changes also occur in this region, external to the brain, including neurogenesis (8). The neural progenitor cells derived from the OE can be isolated from humans (9) and are considered a source of neural progenitor cells (10). In the OE, new neurons are generated from globose basal cells, which proliferate, expressing different transcription factors such as Sox2/Pax6 (stem-like cells), or MASH1 (transit-amplifying progenitors) and NeuroD1 or neurogenin1 (immediate precursor cells). Also, the non integrin laminin receptor precursor protein is expressed on the olfactory stem and progenitor cells (11). Also, hNPCs-OE can produce the brain-derived neurotrophic factor (BDNF) (12), vascular endothelial growth factor (13), interleukin-6 (IL-6), interleukin-8 (IL-8), thrombospondin-1 (THBS1), monocyte chemoattractant protein-1 (MCP-1) and but not limited to, tissue inhibitor of metalloproteinases-1 (TIMP-1) (14).

Numerous studies underscore the significance of assessing the OE in these disorders. For instance, MDD correlates with losing of olfactory function (15). Similarly, individuals with BPD may experience olfactory function alterations (16). In addition, it is suggested that the nasal olfactory epithelium is a dynamic marker for monitoring changes in the central nervous system after therapeutic interventions (17). For instance, the administration of thiamphenicol produced an increased expression of the glutamate transporter EAAT2 in mice and humans who received the drug. Therefore, the OE has been proposed as an area in which the beneficial effects of pharmacological interventions can be monitored (17). Moreover, the alterations in measurable proteins in the OE revealed that the protein SMAD5 (*mothers against decapentaplegic homolog 5)* is a biomarker associated with a cognitive deficit in schizophrenia (18). In another study in patients with bipolar disorder, an association was found in mRNA levels of *glycogen synthase kinase-3* (*GSK3β)* and collapsin response mediator protein 1 (*CRMP1)* with disease status and the severity of mood symptoms. In the same study, the authors found a lithium treatment-associated downregulation of CRMP1 expression predictive of decreases in both manic and depressive symptoms (19).

Thus, we aimed to analyze the presence -and quantify the concentrations- of proteins acting as soluble factors derived from hNPCs-OE of subjects diagnosed with MDD or BPD. Then, we hypothesized that hNPCs-OE of MDD or BPD differentially secreted soluble factors.

## 2 Methods

### 2.1 Participants

A total of 35 subjects were recruited, divided into three groups: 14 subjects with MDD, 14 subjects with BPD, and 7 HC. MDD and BPD patients were recruited from the outpatient service of the National Institute of Psychiatry Ramón de la Fuente Muñiz, where they received the diagnosis from a trained psychiatrist. Controls were invited by snowball sampling from university students and were evaluated by the same psychiatrist to rule out the presence of any psychiatric disorder. All the participants were evaluated according to the Diagnostic and Statistical Manual of Mental Disorders Fifth Edition (DSM-5) to confirm the primary diagnosis or to exclude diagnoses in the controls. The diagnosis of BPD was made by a trained psychiatrist based on the DSM-5 criteria. If the subject met the diagnostic criteria for BPD, then a Structured Clinical Interview for Personality Disorders (SCID-II) was performed. In the three groups, we measured the severity of the depressive symptoms with the Hamilton Depression Rating Scale (HDRS). Then, they were accompanied to the laboratory of neurogenesis for olfactory epithelium sampling. Subjects were excluded if any of the following criteria were found: habitual tobacco use during the last six months, subjects at suicidal risk, receiving treatment with antidepressants that are not from the family of selective serotonin reuptake inhibitors, and who suffered from uncontrolled medical conditions (hypertension, diabetes, rheumatic diseases). The study was performed under the approval of the ethics committee of the National Institute of Psychiatry “Ramón de la Fuente Muñiz, and all subjects gave their written informed consent (Approved number CEI/C/077/2016).

### 2.2 Isolation and characterization of olfactory epithelial cells

**Figure 1:**
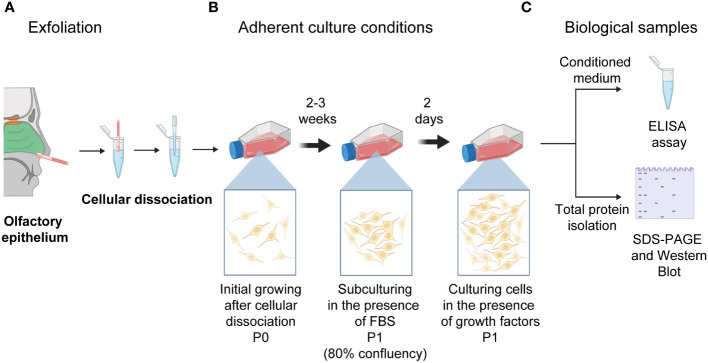
Schematic drawing of the nasal cavity to isolate human neural progenitor cells derived from the olfactory epithelium after exfoliation of the lateral turbinate. **(A)** Exfoliation of the olfactory epithelium and the dissociation of cells is represented. **(B)** Cells were left to grow in adherent culture conditions till reaching confluency (P0). **(C)** Thus, cells were passed to a new culture flask (P1). Once they reached 80% confluency, cells were switched to a medium supplemented with growth factors (20 mg/ml of basic fibroblast and epidermal growth factors). Cells were cultured for two days to collect the conditioned medium and intracellular proteins used for ELISA quantifications and protein level determination after electrophoresis and transferring. Illustrations created with BioRENDER (https://www.biorender.com/).

As previously reported, human neural progenitor cells derived from the olfactory epithelium were obtained (9, 12, 20). Briefly, exfoliated cells from the anterior region of the medial-lateral turbinate were obtained with a special brush and circular movements to get cells from the lateral wall of the nasal cavity and septum. Before nasal exfoliation, participants provided written informed consent for all procedures. Cells were harvested in Dulbecco’s Modified Eagle’s Medium containing F-12 (DMEM/F-12) (Gibco, Life Technologies, NY, USA) supplemented with 10% fetal bovine serum (FBS) (Gibco, Life Technologies), 4 mM L-glutamine (Gibco, Life Technologies), 100 g/ml streptomycin and 100 IU/ml penicillin (Gibco, Life Technologies) and were dissociated mechanically. Dissociated cells were cultured in fetal bovine serum (Gibco, Life Technologies) -supplemented DMEM/F-12 until we observed the cells’ constant expansion. Thus, hNPCs-OE were passed and subcultured with serum-supplemented DMEM/F-12. Two days before the cells were harvested, hNPCs-OE were cultured in the presence of growth factors (20 ng/ml human epidermal growth factor and human fibroblast growth factor 2 with 2% B27). The conditioned medium was collected after 48 hours and centrifuged to eliminate cellular debris. All quantifications by ELISA were performed on cells from passage 1. The conditioned medium was frozen at -80°C (**Figure 1**) (14).

### 2.3 Quantifications of proteins in the conditioned medium of hNPCs-OE

Proteins identified in our previous study (14) were quantified in the conditioned medium of all the cells isolated from the participants in the present study. The levels of interleukin-6, interleukin-8, thrombospondin-1, monocyte chemoattractant protein-1 (MCP-1), tissue inhibitor of metalloproteinase-1 (TIMP-1), and BDNF were determined with ELISA kits (R&D Systems, MI, USA) following the manufacturer instructions. The 96-well plates were read in an ELISA reader of Promega (Glomax Discover) at a wavelength of 450 nm.

### 2.4 Western blot

After collection of the conditioned medium, hNPCs-OE were lysed with RIPA buffer (1X PBS, 0.1% SDS, 1% NP40, 0.5% sodium deoxycholate, 0.24 mg/ml AEBSF, 8 mg/ml aprotinin, 10 mg/ml leupeptin, 4 mg/ml pepstatin, 5 mM benzamidine, 20 mM glycerophosphate, 10 mM NaF, 1 mM Na3VO4, 1 mM EDTA and 1 mM EGTA; Sigma-Aldrich). The total protein content was quantified using a Bradford assay (Abcam, MA, USA). Proteins were separated in an SDS-PAGE system (4 to 12%) and transferred to nitrocellulose paper. The membrane was blocked with 5% of non-fat milk, and proteins were identified with primary antibodies against mouse anti-67kDa laminin receptor (1:3000, Abcam), rabbit anti-Sox2 (1:1000, Sigma-Aldrich), rabbit anti-MASH1 (1:1500, Santa Cruz Immunoresearch, TX, USA), rabbit anti-tubulin beta-III (1:1000, Promega). Beta-actin was used as a loading control (1:2000; Abcam) (**Supplementary Figure 1**). Proteins were visualized with the Millipore-enhanced chemiluminescence detection system in a ChemiDoc™ Touch System (Bio-Rad, Ciudad de México, México). After the identification of every protein, membranes were stripped and exposed to the enhanced chemiluminescence detection system to assure that the previous reaction was eliminated. The densitometric analysis was performed with Image Lab software (Bio-Rad).

### 2.5 Statistical analysis

The descriptive statistics, inference tests, and data visualization in graphs and tables were carried out using the software RStudio v2022.07.1 + 554.pro3. Demographic and clinical data were compared using the chi-square test. Protein levels and ELISA results were tested for normality by the Shapiro–Wilk test and subsequently analyzed by one-way analysis of variance or by Kruskal Wallis one-way analysis of variance on ranks for the three groups. Pairwise comparisons were performed by the Wilcoxon rank sum test (p-value adjustment method: Bonferroni). A Spearman correlation test performed a linear regression between protein measurements and depression score or BPD; then, the results were plotted into a correlation matrix.

## 3 Results

### 3.1 Participants show differences in the Hamilton depression rate score

The demographic and clinical characteristics of the participants are shown in **Table 1**. The patients in the depression group were in a slightly older age range. There was no significant difference between groups regarding sex or marital status. Regarding concurrent health conditions, 50% or more of the sample in each group had no history of any known medical problem. Regarding other mental health conditions, most BPD subjects also had symptoms of depression; both groups had similar proportions of generalized anxiety disorder and panic disorder. The HDRS scores were high in the depression group, intermediate in the BPD group, and low in the control group **Table 1**. Furthermore, we wish to highlight that in our assessment, symptoms in patients with BPD were of short duration and more closely associated with the affective instability characteristic of BPD. In contrast, patients in the MDD group experienced symptoms for a period exceeding two weeks with significant dysfunction associated with depression, which is a key diagnostic criterion for MDD according to the DSM-5.

**Table 1:** Demographic and clinical data.

Variable	Control (N=7) N (%)	MDD (N=14) N (%)	BPD (N=14) N (%)	p Value
Age (Mean ± SD)	22.7 ± 3.1	34.8 ± 13.5	26.4 ± 6.7	0.019
Sex				0.166
- F	6 (85.7%)	8 (57.1%)	16 (85.7%)	
- M	1 (14.3%)	6 (42.9%)	3 (14.3%)	
Marital status				0.089
- Single	7 (100%)	10 (71.4%)	13 (92.9%)	
- Married	0 (0.0%)	4 (28.6%)	0 (0.0%)	
- Divorced	0 (0.0%)	0 (0.0%)	1 (7.1%)	
Socioeconomic status				0.104
- High	3 (42.9%)	2 (14.3%)	1 (7.1%)	
- Low	0 (0.0%)	4 (28.6%)	7 (50.0%)	
- Middle	4 (57.1%)	8 (57.1%)	6 (42.9%)	
Medical comorbidity				0.54
- Asthma	0 (0.0%)	0 (0.0%)	1 (7.1%)	
- Migraine	0 (0.0%)	0 (0.0%)	1 (7.1%)	
- HTN	0 (0.0%)	1 (7.1%)	0 (0.0%)	
- Hyperprolactinemia due to pituitary adenoma	0 (0.0%)	0 (0.0%)	1 (7.1%)	
- Obesity I	0 (0.0%)	1 (7.1%)	0 (0.0%)	
- Obesity II	0 (0.0%)	1 (7.1%)	0 (0.0%)	
- Obesity III	0 (0.0%)	0 (0.0%)	1 (7.1%)	
- Overweight	0 (0.0%)	2 (14.3%)	0 (0.0%)	
- Polycystic Ovary Syndrome	0 (0.0%)	0 (0.0%)	1 (7.1%)	
- Vitiligo	0 (0.0%)	0 (0.0%)	1 (7.1%)	
- No known medical condition	7 (100.0%)	9 (64.2%)	7 (50.0%)	
Psychiatric comorbidity				<0.001
- substance use disorder	0 (0.0%)	1 (7.1%)	0 (0.0%)	
- CSS	0 (0.0%)	2 (14.2%)	0 (0.0%)	
- GAD	0 (0.0%)	5 (35.5%)	3 (21.3%)	
- MDD	0 (0.0%)	14 (100.0%)	10 (71.4%)	
- ADHD	0 (0.0%)	1 (7.1%)	0 (0.0%)	
- Panic Disorder	0 (0.0%)	2 (14.2%)	3 (21.4%)	
HDRS score (Mean ± SD)	8.1 ± 4.6	19 ± 6.6	10.8 ± 5.2	<0.001
BS Score SCID-II (Mean ± SD)	4.9 ± 3.0	NA	11.4 ± 1.9	<0.001

MDD, Major Depressive Disorder; BPD, Borderline Personality Disorder; CSS, Caregiver Stress Syndrome; GAD, Generalized Anxiety Disorder; HDRS, Hamilton depression rating scale; HPV, Human Papillomavirus; HTN, Hypertension; ADHD, Attention deficit hyperactivity disorder; SCID-II, Structured Clinical Interview For DSM-IV; BS, Borderline Subscale. NA, Not Applied.

### 3.2 Human neural progenitor cells show modifications in the expression of characteristic proteins of progenitor cells.

**Figure 2:**
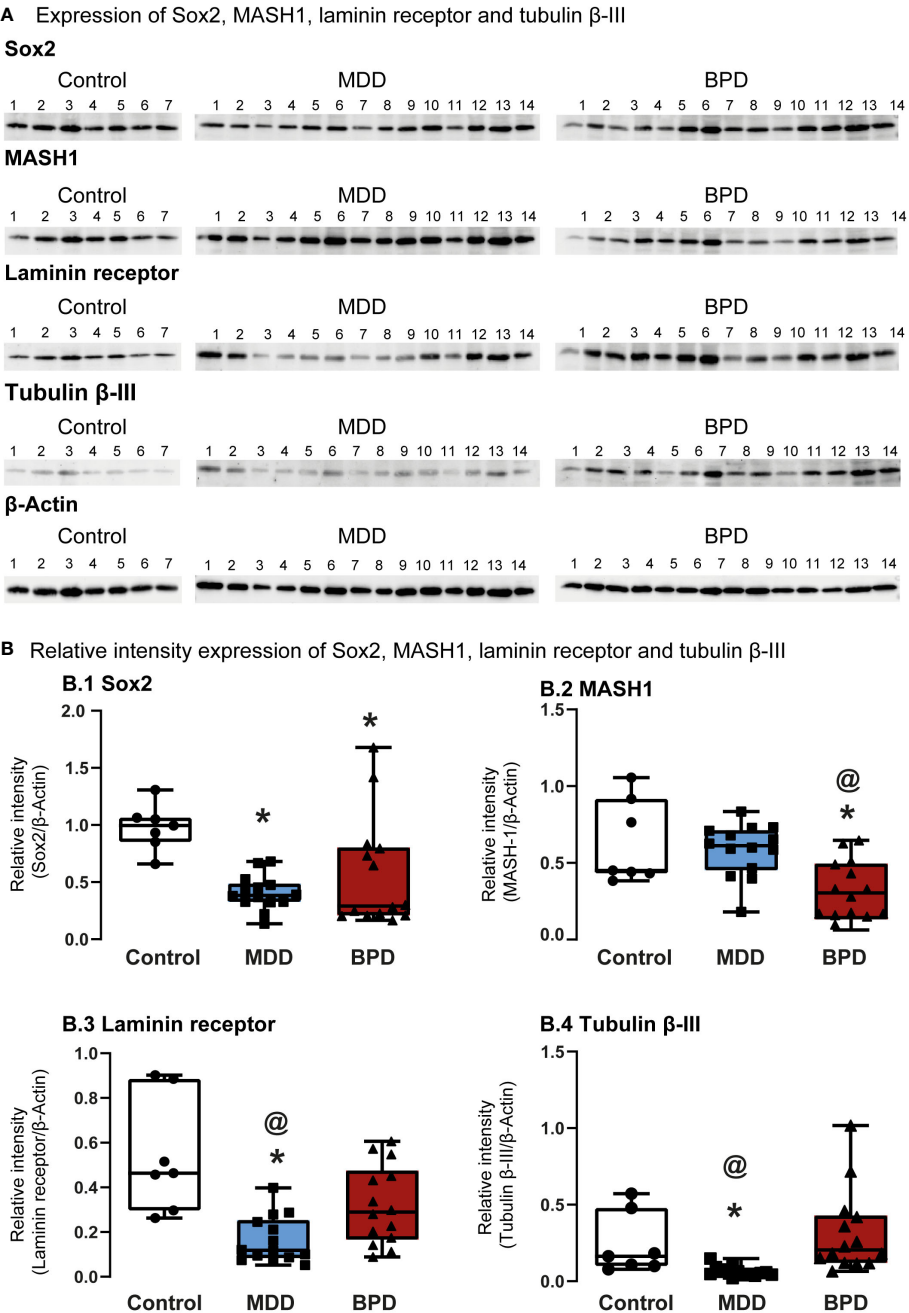
Protein markers expressed in human neural progenitor cells derived from the olfactory epithelium. **(A)** Representative immunoblots of Sox2 **(A.1)**, MASH-1 **(A.2)**, laminin 67D receptor **(A.3)**, and tubulin beta III **(A.4)**. Blots include proteins of human neural progenitor cells derived from the olfactory epithelium of the control (n=7), major depressive disorder (MDD, n=14) and borderline personality disorder (BPD, n=14). **(B)** Histograms show protein level quantification of Sox2 **(B.1)**, MASH-1 **(B.2)**, laminin 67D receptor **(B.3)**, and tubulin beta III **(B.4)** normalized against actin-beta as a loading control. Data were tested for normality by the Shapiro–Wilk test and subsequently analyzed by one-way analysis of variance or by Kruskal Wallis one-way analysis of variance on ranks for the three groups. Pairwise comparisons were performed by the Wilcoxon rank sum test (p-value adjustment method: Bonferroni). Significant results were found with a p-value <0.05. Asterisks (*) indicate significant differences against the control group. The symbol at (@) shows a significant difference between MDD and BPD. Results indicate mean ± standard error of the mean (S.E.M.).

Western Blot assessed protein levels of progenitor cells’ characteristic markers (**Figure 2A**). The level of Sox2, a transcription factor that is a persistent marker for multipotential neural stem cells, showed a significant decrease in hNPCs-OE of MDD and BPD cells compared with the control group (q=3.027, p=0.007; q=2.92, p=0.01; H=10.63, d.f. = 2, p=0.005; **Figure 2B.1**). Interestingly, the comparison of MDD and BPD almost reached significance (q=3.83, p=0.057). The protein levels of MASH1 (**Figure 2B.2**), also known as Ascl1, that is expressed in transit-amplifying progenitors with a limited capacity for expansive proliferation (11), showed a significant decrease in BPD compared with control (p=0.005) and MDD (p=0.004; F=2,32 = 8.45, p=0.001) groups. A similar pattern to Sox2 was seen in the levels of the 67 kDa laminin receptor in MDD or BPD compared with the control group (q=6.69, p=0.0001; q=3.93, p=0.02; H=14.26, d.f. = 2, p=0.001; **Figure 2B.3**), a protein expressed in olfactory stem and progenitor cells (11). Tubulin beta-III (**Figure 2B.4**) in MDD hNSPCs-OE showed decreased levels compared with the control (q=2.96, p=0.001) and BPD (q=4.31, p<0.0001)(H=20.36, d.f. = 2, p=0.001).

### 3.3 The conditioned medium of human neural progenitor cells contains increased levels of IL-6, IL-8, Thrombospondin, MCP-1, and BDNF

**Figure 3:**
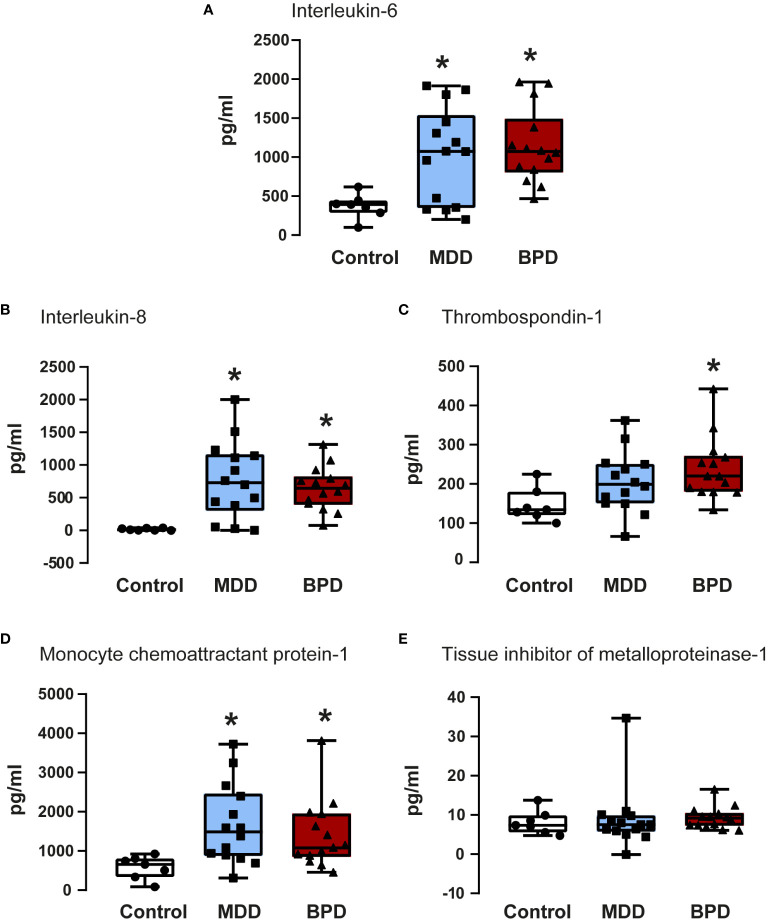
Quantifications of soluble factors in the conditioned medium of human neural progenitor cells derived from the olfactory epithelium. Quantification of proteins released to the conditioned medium by human neural progenitor cells derived from the olfactory epithelium of the control (n=7), major depressive disorder (MDD, n=14), and borderline personality disorder (BPD, n=14) corresponded to interleukin-6 **(A)**, interleukin-8 **(B)**, thrombospondin-1 **(C)**, monocyte chemoattractant protein-1 **(D)** and the tissue inhibitor of metalloproteinase-1 **(E)** are shown. Data were tested for normality by the Shapiro–Wilk test and subsequently analyzed by one-way analysis of variance or by Kruskal Wallis one-way analysis of variance on ranks for the three groups. Pairwise comparisons were performed by the Wilcoxon rank sum test (p-value adjustment method: Bonferroni). Significant results were found with a p-value <0.05. Asterisks (*) indicate significant differences in MMD or BPD against the control group. Results indicate mean ± standard error of the mean (S.E.M.).

A previous study of our group showed that among the proteins found in the conditioned medium of hNPCs-OE exist IL-6, IL-8, thrombospondin-1, MCP-1, and TIMP-1 (14). Thus, we quantified the concentrations of the proteins mentioned above in the conditioned medium of hNPCs-OE of MDD, BPD, and HC participants, respectively (**Figure 3**).

IL-6 showed a significant contrast between the three groups (**Figure 3A**). Subsequently, a pairwise comparison with the Wilcoxon test was performed to assess whether there were statistically significant differences between groups; the distinction was important between the control group with the BPD group (p=0.0001) and with the MDD group (p=0.05), with no difference between the BPD and MDD groups (p=0.6). Similarly, protein quantifications showed a difference in IL-8 concentration (**Figure 3B**) in the global analysis of three groups, presenting an essential difference between the control group and the BPD group (p<0.0001) and the MDD group (p=0.002) but not between the MDD and BPD groups (p = 0.7). For thrombospondin-1 (**Figure 3C**), there was an overall significant difference (p=0.02), which was only consistent with a difference between the control group and BPD (p=0.01) but not for the control vs. MDD (p=0.08) or MDD vs. BPD (p = 0.2). Regarding MCP1 (**Figure 3D**), the global difference showed a p equal to 0.009, the pairwise comparison between the control group with BPD (p=0.01) and with MDD (p=0.009) being significant, with no difference between the MDD and BPD groups (p = 0.6). Finally, for the TIMP1 protein (**Figure 3E**), there was no difference between the global comparison (p = 0.4) or between groups (control vs. BPD p=0.5, control vs. MDD p=0.9, BPD vs. MDD p=0.5). Similar observation occurred after BDNF quantification which did not show statistically significant differences among the groups (**Table 2**).

**Table 2:** BDNF Values.

Variable	Control (N=7) N (%)	MDD (N=14) N (%)	BPD (N=14) N (%)	p Value
BDNF (pg/ml)	0.299 ± 0.046	0.271 ± 0.064	0.367 ± 0.080	0.59

Data of BDNF quantification in the conditioned medium of hNPCs-OE of control, MDD, and BPD are shown. Results indicate the mean ± standard error of the mean. Statistical analysis does not indicate significant differences among the groups.

### 3.4 Correlations

**Figure 4:**
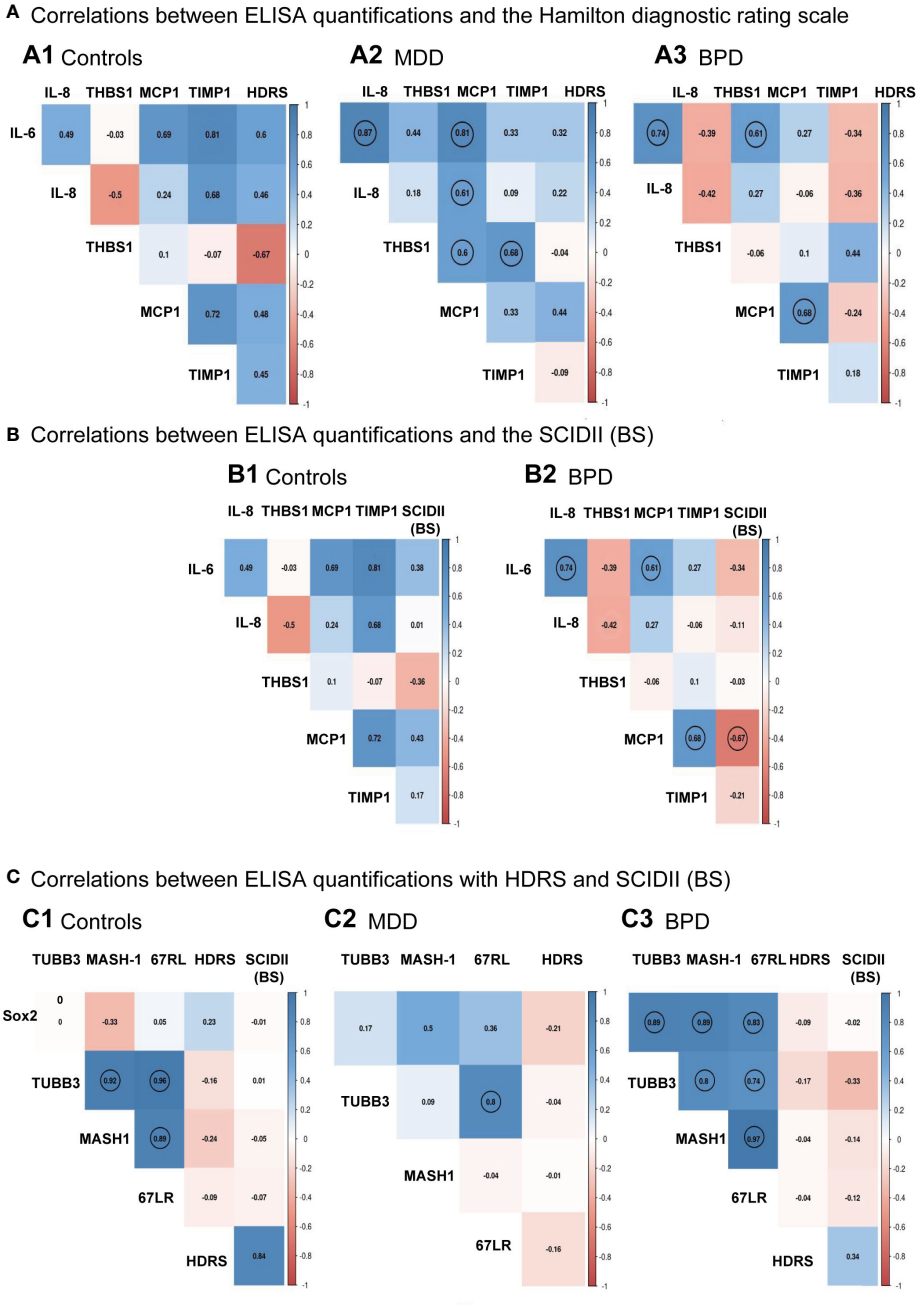
Individual correlation matrices with clinical scales to diagnose major depressive disorder or borderline personality disorder. **(A)** Correlation among ELISA quantifications and the Hamilton Depression Rating Scale (HDRS) for the control group **(A.1)**, major depressive disorder (MDD, **A.2**), and borderline personality disorder [BPD, **(A.3)**], respectively. **(B)** Correlation among ELISA quantifications and the Structured Clinical Interview for Personality Disorders (SCID-II) scale for the control group **(B.1)** and borderline personality disorder [BPD, **(B.2)**], respectively. **(C)** Correlation among protein levels quantifications done in the human neural progenitor cells derived from the olfactory epithelium and the HDRS and SCID-II scales for the control group **(C.1)**, major depressive disorder [MDD, **(C.2)**] and borderline personality disorder [BPD, **(C.3)**], respectively. Matrices showed significant relationships after Pearson correlation analysis with a p<0.05 indicated with a circle for the control group (n=7), MDD (n=14), and BPD (n=14). Pairwise comparisons were performed by the Wilcoxon rank sum test (p-value adjustment method: Bonferroni). A Spearman correlation test performed a linear regression between protein measurements and depression score or BPD; then, the results were plotted into a correlation matrix.

The next step was to evaluate if there was a correlation between the HDRS scores and each protein quantified by ELISA for each group. Results are shown in **Figure 4A**. No significant correlation was found between the HDRS scores and the proteins quantified for the three groups, while a correlation was found among various cytokines (**Supplementary Table 1**). For instance, hNSPCs-OE in MDD, IL-6 positively correlated with IL-8 (r=0.87, p<0.001) and MCP-1 (r=0.81, p<0.001). The IL-8 correlated with MCP-1 (r=0.27, p=0.019). Also, Thrombospondin-1 correlated with MCP-1 (r=0.60, p=0.022) and TIMP-1(r=0.68, p=0.007). In BPD, IL-6 correlated with IL-8 (r=0.74, p=0.002) and MCP-1 (r=0.61, p=0.02). The MCP-1 with TIMP-1 (r=0.67, p=0.007).

Correlation analysis was also performed between the levels of cytokines with the scores of the borderline personality disorder subscale of the SCID-II (**Figure 4B**; **Supplementary Table 2**), where it is shown that there was only a significant negative association between the SCID-II (BS) score with MCP-1 (r=-0.66, p = 0.008). We see the same correlations indicated in panel A3 of **Figure 4** for correlations among proteins.

We also evaluated whether there was a correlation between the scores of the Hamilton depression rating scale and the borderline section of the SCID-II (BS) scale with levels of protein markers quantified by Western blot in hNPCs-OE. We did not find a correlation between these proteins and the clinical scales (**Figure 4C**, **Supplementary Table 3**). However, in HC hNPC-OE protein marker levels showed a positive correlation in tubulin beta-III with MASH-1 (r=0.82, p=0.02) and the laminin 67 kDa protein (r=0.96, p<0.001). In the case of MASH-1, it correlated with the laminin 67 kDa protein (r=0.89, p=0.006). For MDD, we only found a positive correlation between the laminin 67 kDa protein and tubulin beta-III (r=0.8, p<0.001). Interestingly, we found positive correlations for BPD among all proteins quantified by Western blot. For instance, Sox2 correlated with tubulin beta-III (r=0.89, p<0.001), MASH-1 (r=0.88, p<0.001) and the laminin 67 kDa protein (r=0.82, p<0.001). For the tubulin beta-III, it correlated with MASH-1 (r=0.97, p<0.001) and the laminin 67kDa protein (r=0.74, p=0.002). Finally, MASH-1 correlated with the laminin 67 kDa protein (r=0.97, p<0.001).

### 3.5 Protein-protein interaction analysis

**Figure 5:**
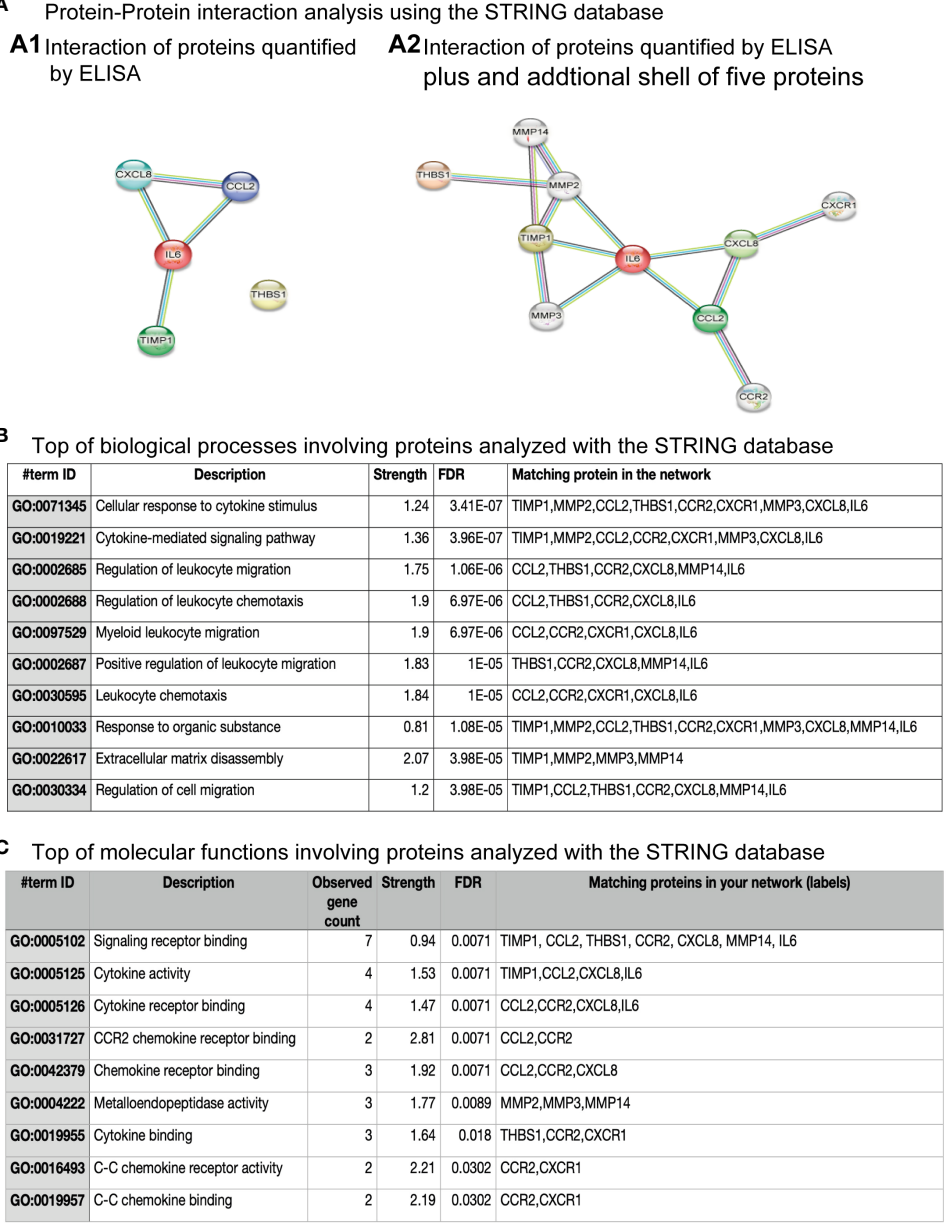
Protein-protein interaction among proteins quantified by ELISA. **(A)** Protein-protein interactions analysis using the STRING database. **(A.1)** shows the interaction among four of the five proteins quantified by ELISA. However, **(A.2)** shows the interaction among proteins quantified by ELISA and after the addition of a sale of five proteins. **(B)** The top biological processes involving proteins analyzed with the STRING database are shown. The inserted table includes the item ID, the description, strength, false discovery rate (FDR), and the matching protein in the network. **(C)** The top molecular functions involving proteins analyzed with the STRING database are shown. The inserted table includes item ID, the description, the number of genes observed, strength, false discovery rate (FDR), and the matching protein in the network.

Thus, we used the STRING database to know protein-protein interactions (PPIs) among the soluble factors quantified in hNPCs-OE of controls, MDD, and BPD groups (**Figure 5**). We used the highest confidence score for PPIs among IL-6, IL-8, thrombospondin-1, MCP-1, and TIMP-1 (0.9). The analysis revealed five nodes with several edges equal to 4 and a PPI enrichment p-value of 6.6x10-5, suggesting that the proteins are partially biologically connected as a group (**Figure 5A.1**). However, after adding a second shell of no more than five interactions, we found ten nodes, 13 number of edges and a PPI enrichment p-value of 0.0046 (**Figure 5A.2**). The top ten of biological processes after gene ontology are shown in panel 5B. Among the biological processes are some of them involved in the cellular response to cytokine stimulus, cytokine-mediated signaling pathways, migration, and chemotaxis. Regarding the molecular functions involving the proteins quantified by ELISA (**Figure 5C**), the top ten are related to signaling receptor binding, cytokine receptor binding, and activity.

## 4 Discussion

This study found that hNPCs-OE differentially expressed protein markers related to their progenitor stage in MDD and BPD. Also, hNPCs-OE showed increased IL-6, IL-8, THBS1, and MCP1 concentrations in MDD and BPD compared with control participants. Interestingly, the importance of these proteins in these psychiatric disorders is based on their implication in their pathogenesis (21–24).

In contrast with prior research, this study aligns with some findings while introducing novel insights. Previous studies have hinted at protein expression differences in MDD and BPD (15, 16), but the exact patterns identified here, particularly concerning Sox2 and MASH1, could be unique. The alterations in Sox2 and MASH1 expression may have functional implications; such changes can potentially influence neural plasticity, neurogenesis, and overall brain function (7, 25), which, in turn, might affect the clinical manifestations of MDD and BPD. The exact nature of this influence remains to be elucidated.

The elevated cytokine levels underscore the potential role of inflammation in MDD and BPD (21–23, 26). This study significantly contributes to the mounting evidence linking inflammation to these disorders. Yet, discerning whether inflammation is a cause or consequence remains complex, with more research needed to isolate the sources of such inflammatory responses. From a clinical perspective, the differential protein expression combined with elevated cytokine levels introduces potential therapeutic targets for MDD and BPD (21–23, 26). However, translating these insights into actionable interventions presents challenges. Normalizing protein expression or modulating cytokine levels could form the basis of new treatments, but these strategies would be in their infancy and require rigorous validation.

While correlations between Hamilton Depressión Rating Scale scores and protein levels were absent, it prompts questions about other potential clinical or symptom measures that might align more closely with these protein changes. Furthermore, protein expression patterns might differ in specific subgroups of MDD and BPD patients, especially those with severe manifestations or resistance to conventional treatments (15). In terms of diagnosis, this study suggests a promising avenue where Sox2, MASH1, and other identified proteins could serve as potential biomarkers for MDD and BPD. These findings could refine diagnostic criteria if corroborated, presenting a more nuanced and biologically informed approach to diagnosing these disorders.

### 4.1 Human neural progenitor cells derived from the olfactory epithelium

The OE-derived cells have gained attention for the study of psychiatric disorders. Also, the OE is easily accessible to isolate some of its cell types, and recent studies support the relevance of obtaining OE cells from living participants (27). Interestingly, the generation of new neurons in the OE follows a serial process involving globose basal stem cells (GBCSTEM) and globose basal multipotent (GBCMPP) cells identified by the expression of the transcription factors Sox2 and Pax6; globose basal transit-amplifying cells (GBCTA-OSN) restricted to a neuronal fate identified by the expression of Sox2, Pax6, and MASH1; globose basal immediate neuronal precursor cells (GBCINP) identified by the expression of neurogenin and NeuroD1 to further decrease their expression during the transition to mature olfactory sensory neurons (For a review see (7)). Previous studies of our group (9, 12, 14) confirmed that cells isolated from the OE showed the expression of nestin and tubulin beta-III, as was previously reported (9). Here, it is important to note that tubulin beta-III is coexpressed with the neuroepithelial stem cell protein nestin (9, 28). However, tubulin beta-III is still expressed, but at lower levels, in differentiated hNPCs-OE (14). Here, hNPCs-OE in healthy controls expressed Sox2, the laminin 67 kDa protein, MASH1, and tubulin beta-III, suggesting that it may correspond to GBCTA-OSN.

Interestingly, the decreased levels of Sox2, the laminin 67 kDa protein, and tubulin beta-III without modifications of MASH1 in hNPCs-OE in MDD suggest that hNPCs-OE stayed in a multipotent stage with the capability to form not only neurons but also astrocytes (personal communication Dr. Adan Hernández-Cortés, Instituto de Neurobiología, UNAM). However, the decreased levels of Sox2, the laminin 67 kDa protein, and MASH1 with a slight increase in tubulin beta-III in hNPCs-OE in BPD suggest that these cells may go from GBCTA-OSN to the GBCINP stage. Thus, the temporal expression of protein markers in hNPCs-OE may suggest differences between MDD and BPD compared with healthy controls. Therefore, more correlations among the protein markers in BPD hNPCs-OE could reflect a more dynamic process than in MDD hNPCs-OE that may impact the generation of newborn neurons. Unfortunately, we could not detect the presence of neurogenin-1 or NeuroD1, protein markers expressed in GBCINP, to confirm the transition of GBCTA-OSN to the GBCINP stage (7).

### 4.2 Soluble factors found in human neural progenitor cells derived from the olfactory epithelium

IL-6 protein showed clear differences between the control group and the clinical groups. IL6 has been identified as one of the central cytokines associated with depression (29, 30); it is also known that one of the main functions of IL6 is to promote the secretion of proinflammatory cytokines leading to systemic inflammation, in addition within the peripheral and Central Nervous System (CNS), IL-6 can act as a neuronal growth factor inducing neurite development and nerve regeneration (31–33). IL-6 also plays an essential role in balancing anti-inflammatory and proinflammatory responses (31–33). There is evidence that circulating levels of IL6 may be increased before the onset of MDD (31–33), and a functional role for IL-6 in stress susceptibility has been proposed (34–38). Clinical studies have also revealed that patients with MDD have increased plasma and serum concentrations of proinflammatory cytokines, including IL-6, compared to healthy controls (39, 40). Also, previous studies showed that elevated levels of proinflammatory cytokines such as IL-6 might affect neurogenesis (38) and neural plasticity (41).

Regarding BPD, the findings are not so consistent; in a clinical trial, it was found that IL-6 levels were not different from controls (41), while in another study in BPD patients comorbid with major depression, increased IL-6 have been found compared to controls (42). In our sample, subjects with BDP showed a difference in IL-6 compared with controls but not with subjects with MDD. This difference suggests that IL-6 measured in the hNPCs-OE may be a promising marker for seeking differences between Control subjects with MDD or BPD. However, the specificity of this protein as a possible marker in hNPCs-OE for these two disorders should be further studied.

A similar finding was presented in IL-8, with an important difference between control and clinical groups. IL-8 is a proinflammatory cytokine produced by many cell types; one of its primary functions is to serve as a neutrophil chemoattractant in the bloodstream. IL-8 is also found in the brain, where it is released from microglia in response to proinflammatory stimuli and may be implicated in various psychiatric diseases. The literature on IL-8 and major depression is inconsistent. Three meta-analyses have demonstrated that people with MDD showed no differences in serum/plasma IL-8 levels compared to people without depression (29, 43, 44). One reason for the lack of significant findings may be the small sample size of these included studies. In a small-scale meta-analysis (including two studies, 38 MDD patients and 114 controls), CSF Levels of IL-8 were significantly increased in MDD patients compared to healthy controls (45). There is less certainty about the role of IL-8 in BDP; in one study, a lower association was found between the genetic expression of IL-8 compared to IL-6 with dissociative symptoms in BPD (46); in another study, similar concentrations were found of IL-8 in young women with MDD comorbid with BPD when compared to a healthy comparator group (47). One possibility is that due to the high phenotypic diversity of BPD (48), the elevation of IL-8 corresponds to the convergence of other inflammatory processes or environmental factors (49) rather than being something typical of BPD. However, in hNPCs-OE, IL-8 may act as a marker for both neuropsychiatric disorders compared with control subjects. Preliminary observations in our group suggest that the level of IL-8 in the conditioned medium of hNPCs-OE in MDD may be a predictor of response to treatment.

MCP-1 (CCL2) is best recognized as a chemoattractant and activator of monocyte/macrophages, T lymphocytes, and dendritic cells. In the central nervous system, MCP-1 and its receptor (CCR2) are widely expressed in astrocytes, microglia, neurons, and neural stem/progenitor cells (50). MCP-1 may be essential in regulating the inflammatory activation state of CNS resident microglia (47). While emerging electrophysiological data suggest a neuromodulatory effect of MCP-1 (51), most reports indicate that MCP-1 is increased in the serum of patients with depression compared to controls (52–54). Also, it has been reported that over-expression of MCP-1 is associated with the severity of depressive symptoms (55).

Regarding MCP-1 in BPD, little literature reports findings of MCP-1 in BPD. A study found increased peripheral levels of this cytokine in subjects with generalized anxiety disorder comorbid with avoidant personality disorder, borderline personality disorder, or obsessive-compulsive personality disorder compared with controls (56). Interestingly, we found a significant negative correlation between MCP-1 in hNPCs-ONE with the SCIDII (BS). Thus, the relevance of this correlation needs further consideration in future studies with a higher sample size. However, the previously mentioned changes were not found when comparing thrombospondin-1 (THBS-1) between controls and subjects with MDD, and there was no critical difference for TIMP-1 and BDNF between the three groups. The literature on thrombospondin-1 and TIMP-1 in MDD and BPD is scarce. However, it is increased in women with depression, but in the same study, no correlation was found between THBS-1 levels with symptoms or antidepressant dose (57). In another study, no association was found between THBS-1 and predictive variables of sexual abuse or symptoms of depression (58). Since THBS-1 is involved in stress and inflammation processes in the CNS (59, 60). It is necessary to study further its role in BPD since various inflammatory alterations have been found in this disorder (61). Regarding BDNF, in the present study, we confirmed that hNPCs-OE secrete BDNF (12). However, the concentrations of BDNF seem to be constant in the conditioned medium of hNPCs-OE independently of the psychiatric entity, at least for MDD and BPD or treatment in MDD (62, 63). This aspect needs to be confirmed by contrasting the effects of several treatments and or with a higher number of participants.

The difference we observed in the control group with the clinical population in the cytokines expressed in hNPCsOE is consistent with the close interrelationship between stress and inflammation. Psychosocial stress or physical illness can activate brain microglia, which increases the secretion of proinflammatory cytokines and, in turn, affects neurogenesis and synaptic plasticity. Under normal conditions, stress-related inflammatory activity is downregulated by the HPA axis through cortisol production. However, when there is prolonged real or perceived social threat or physical danger, glucocorticoid resistance can develop, leading to excessive inflammation that increases a person’s risk of developing various disorders, including MDD, especially if the activation of these pathways is prolonged (64, 65). In addition, increased secretion of proinflammatory cytokines can reduce the bioavailability of neurotransmitters such as serotonin (65, 66).

### 4.3 Correlations

Regarding the results in measuring the intensity of depressive symptoms, we found these were low in controls, intermediate in BPD, and high in MDD. On the other hand, there is no correlation between the scores of the HDRS and the levels of inflammatory cytokines. These findings contrast with previous reports in which it has been seen that peripheral IL-6 levels can vary according to the subtype of depression (67). Another critical factor is exposure to adverse childhood experiences, associated with an increased risk of developing MDD later in life. One study has shown that MDD patients with adverse childhood experiences showed significantly higher IL-6 concentrations compared to healthy controls and MDD patients without negative childhood experiences (68). A study of 732 Korean elders found that depression at baseline was significantly associated with higher serum IL-8 levels; in addition, incident depression was significantly associated with increases in IL-8 levels during the 2-year follow-up (69).

In contrast, in a study with a community sample of 201 adolescents tested for inflammatory proteins and followed up for depression, higher IL-8 predicted lower depressive symptoms than 31 months in men (70). Furthermore, our data revealed a negative correlation between MCP-1 levels and the SCID-II (BS) scores. This finding might indicate that, although individuals with BPD generally exhibit elevated basal levels of MCP-1 compared to controls, there appears to be an inverse relationship between symptom severity and MCP-1 concentration within the BPD sample. It is crucial to note that cytokine measurements were taken from serum samples in the studies above. Furthermore, it’s essential to evaluate, using larger samples, whether there’s a correlation between these protein levels and clinical symptoms.

### 4.4 Potentiality and limitations of hNPCs-OE as distinctive biomarkers in BPD and depression

The identification of reliable biomarkers in disorders such as MDD and BPD has been a constant challenge in psychiatric research. While specific markers, such as IL-6 and IL-8, have been repeatedly associated with these conditions, there is significant variability in findings between studies, complicating their clinical use. In this study, we have presented data suggesting that hNPCs-OE might be a promising source of biomarkers for these disorders. A key advantage of this approach is the potential relationship of these biomarkers with neurogenic processes, which could offer a unique window into the underlying neuronal dynamics in MDD and BPD (17). Additionally, hNPCs-OE overcomes some of the limitations of serum biomarker measurements, which can be affected by a wide range of external factors (15). Our results shed light on the interaction and potential differences in biomarkers associated with MDD and BPD. Both human neural progenitor cells and proteins in the conditioned medium showed distinctive modifications depending on the study group. However, it is essential to highlight some inconsistencies and key differences.

On the one hand, while the depression group showed higher scores on the HDRSe scale, their levels of specific proteins, such as Sox2 and Tubulin beta-III, significantly decreased compared to the control and BPD groups. Moreover, although IL-6 and IL-8 indicated significant differences between the control, MDD, and BPD groups, there was no clear distinction between the MDD and BPD groups. This overlap suggests the complexity of discerning these specific biomarkers for each disorder. It is crucial to consider these findings and the observed variations when interpreting the clinical relevance of these potential biomarkers. The correlations between HDRS scores and proteins and protein-protein interactions further reinforce the complexity and the need to approach these findings with careful interpretation.

### 4.5 Limitations

The study’s significant limitations include a small sample size and the lack of plasma cytokine levels for assessment. The absence of substantial differences between the BPD and MDD groups may be attributed to similar comorbidities. In addition, we recognize that only one group has pharmacological treatment, which is a study limitation. It is important to note that future studies should aim to evaluate the pharmacological role in these contexts. A detailed analysis of the impact of specific medications on the studied variables could provide more nuanced insights into the interaction between pharmacotherapy and the physiological or psychological aspects of BPD and MDD. This approach would enhance the understanding of how medication influences the outcomes of these disorders, thereby contributing to more tailored and effective treatment strategies.

## 5 Concluding remarks

We observed differences in three inflammatory cytokines when contrasting control groups with those of MDD and BPD. This suggests that olfactory epithelial cells might be promising candidates for deriving neural markers in psychiatric conditions. Nonetheless, more extensive studies encompassing larger samples and diverse clinical metrics are essential to ascertain the viability of this method as a diagnostic biomarker for mental disorders (25). Nevertheless, our analysis indicated that hNPCs-OE differentially exhibited protein markers, aligning with their progenitor stage in MDD and BPD. This highlight potential disparities in these cells’ capacities to foster new neurons.

Furthermore, hNPCs-OE registered heightened concentrations of IL-6, IL-8, thrombospondin-1, and MCP1 in MDD and BPD groups, positioning the soluble factors from these cells as potential biological indicators to distinguish among MDD, BPD, and control subjects. Notably, the significance of these proteins in these disorders is underscored by their involvement in pathogenesis (21). As a relevant point, numerous risk elements for MDD, ranging from familial to medical domains, correlate with alterations in cytokine production or signaling (71–73).

## Data availability statement

The raw data supporting the conclusions of this article will be made available by the authors, without undue reservation.

## Ethics statement

The studies involving humans were approved by Ethics committee of the National Institute of Psychiatry “Ramón de la Fuente Muñiz (Approved number CEI/C/077/2016). The studies were conducted in accordance with the local legislation and institutional requirements. The participants provided their written informed consent to participate in this study.

## Author contributions

AD: Data curation, Formal analysis, Investigation, Methodology, Software, Writing – original draft. FV: Data curation, Formal analysis, Investigation, Methodology, Writing – review & editing. GR: Conceptualization, Funding acquisition, Project administration, Supervision, Visualization, Writing – original draft, Writing – review & editing. MF: Conceptualization, Supervision, Validation, Writing – review & editing. PR: Formal analysis, Investigation, Methodology, Writing – original draft. JG: Conceptualization, Supervision, Writing – review & editing. RS: Validation, Visualization, Writing – review & editing.

## Supplementary material

Supplementary Figure 1 Western blots of protein markers expressed in human neural progenitor cells derived from the olfactory epithelium. The figure shows Sox2, MASH1, laminin 67D receptor (Laminin R.), tubulin beta III, and actin-beta immunoblots, which correspond to the control group (left column), participants diagnosed with major depressive disorder (MDD) or borderline personality disorder (BPD). Asterisks (right side) indicate the proteins with molecular weights previously reported in **Figure 2**.
