# Supplementary figures and images for: Human olfactory neural progenitor cells reveal differences in IL-6, IL-8, thrombospondin-1, and MCP-1 in major depression disorder and borderline personality disorder
Source: Front Psychiatry. 2024 Apr 9;15:1283406. doi: 10.3389/fpsyt.2024.1283406 (PMC11035822; doi:10.3389/fpsyt.2024.1283406)

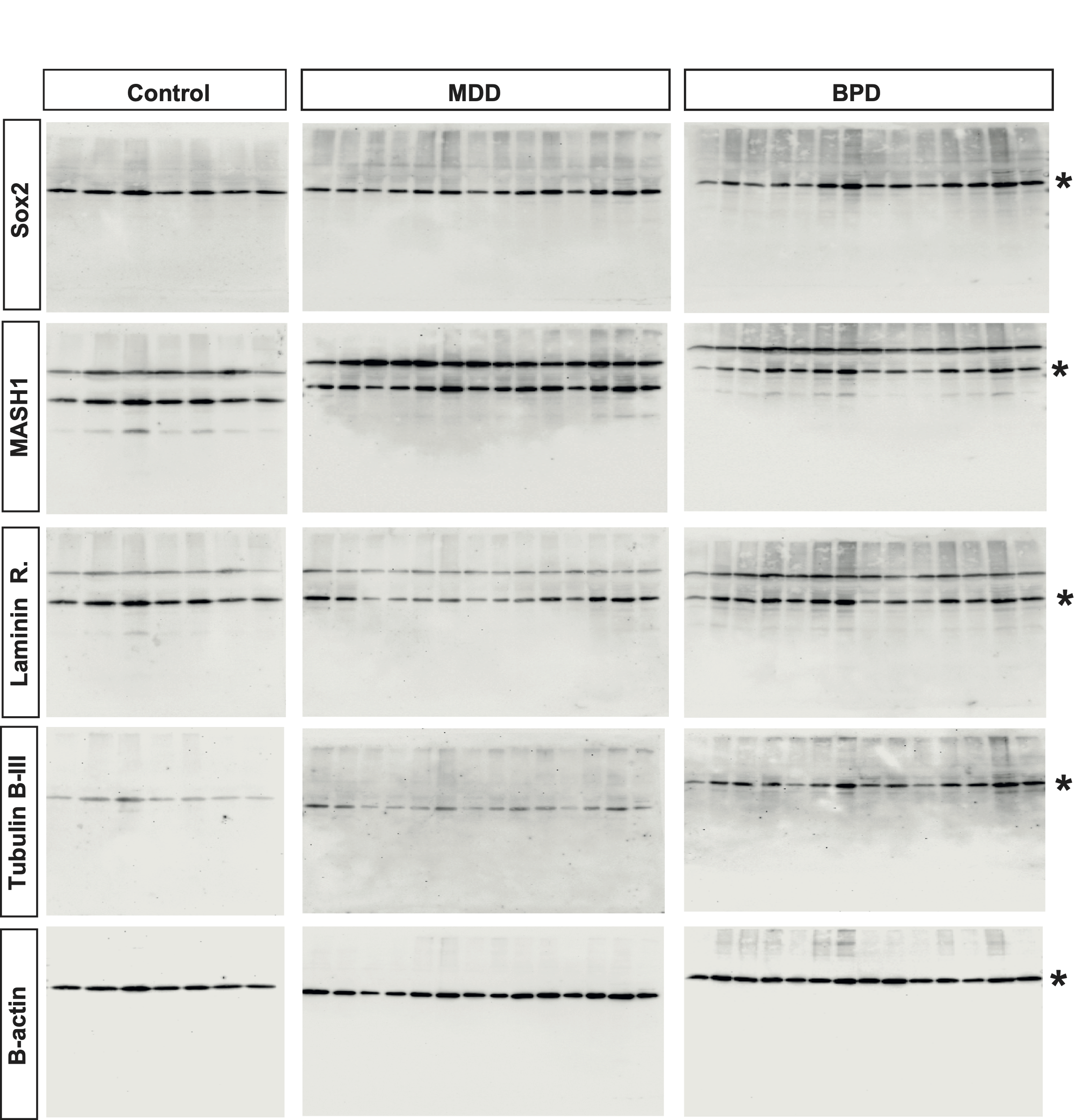

Supplement: Supplementary Figure 1 — Western blots of protein markers expressed in human neural progenitor cells derived from the olfactory epithelium. The figure shows Sox2, MASH1, laminin 67D receptor (Laminin R.), tubulin beta III, and actin-beta immunoblots, which correspond to the control group (left column), participants diagnosed with major depressive disorder (MDD) or borderline personality disorder (BPD). Asterisks (right side) indicate the proteins with molecular weights previously reported in Figure 2 . [file Image_1.tiff]
